# Influence of Genetic Variation in *PDE3A* on Endothelial Function and Stroke

**DOI:** 10.1161/HYPERTENSIONAHA.119.13513

**Published:** 2019-12-23

**Authors:** Matthew Traylor, Ali Amin Al Olama, Leo-Pekka Lyytikäinen, Sandro Marini, Jaeyoon Chung, Rainer Malik, Martin Dichgans, Mika Kähönen, Terho Lehtimäki, Christopher D. Anderson, Olli T. Raitakari, Hugh S. Markus

**Affiliations:** 1 From the Stroke Research Group, Department of Clinical Neurosciences, University of Cambridge, United Kingdom (M.T., A.A.A.O., H.S.M.); 2 William Harvey Research Institute, Barts and The London School of Medicine and Dentistry, Queen Mary University of London, United Kingdom (M.T.); 3 Department of Clinical Chemistry, Fimlab Laboratories, Tampere, Finland (L.-P.L., T.L.); 4 Department of Clinical Chemistry (L.-P.L., T.L.), Finnish Cardiovascular Research Center Tampere, Faculty of Medicine and Health Technology, Tampere University, Finland; 5 Department of Clinical Physiology (M.K.), Finnish Cardiovascular Research Center Tampere, Faculty of Medicine and Health Technology, Tampere University, Finland; 6 Center for Genomic Medicine, Massachusetts General Hospital, Boston (S.M., J.C., C.D.A.); 7 Division of Neurocritical Care and Emergency Neurology, Department of Neurology (S.M., C.D.A.), Massachusetts General Hospital, Boston; 8 Department of Neurology, McCance Center for Brain Health (C.D.A.), Massachusetts General Hospital, Boston; 9 Program in Medical and Population Genetics, Broad Institute, Cambridge, MA (S.M., J.C., C.D.A.); 10 Institute for Stroke and Dementia Research, Klinikum der Universität München, Ludwig-Maximilians-Universität München, Germany (R.M., M.D.); 11 Munich Cluster for Systems Neurology, Germany (M.D.); 12 Department of Clinical Physiology, Tampere University Hospital, Finland (M.K.); 13 Department of Clinical Physiology and Nuclear Medicine, Turku University Hospital, Finland (O.T.R.); 14 Research Centre of Applied and Preventative Cardiovascular Medicine, University of Turku, Finland (O.T.R.).

**Keywords:** cyclic nucleotide phosphodiesterases, type 3, genetics, genome-wide association study, stroke, vascular endothelium

## Abstract

We aimed to characterize the genetics of endothelial function and how this influences risk for cardiovascular diseases such as ischemic stroke. We integrated genetic data from a study of ultrasound flow-mediated dilatation of brachial artery in adolescents from ALSPAC (Avon Longitudinal Study of Parents and Children; n=5214) with a study of ischemic stroke (MEGASTROKE: n=60 341 cases and 452 969 controls) to identify variants that confer risk of ischemic stroke through altered endothelial function. We identified a variant in *PDE3A* (Phosphodiesterase 3A), encoding phosphodiesterase 3A, which was associated with flow-mediated dilatation in adolescents (9–12 years of age; β[SE], 0.38 [0.070]; *P*=3.8×10^−8^) and confers risk of ischemic stroke (odds ratio, 1.04 [95% CI, 1.02–1.06]; *P*=5.2×10^−6^). Bayesian colocalization analyses showed the same underlying variation is likely to lead to both associations (posterior probability, 97%). The same variant was associated with flow-mediated dilatation in a second study in young adults (age, 24–27 years; β[SE], 0.47 [0.23]; *P*=0.047) but not in older adults (β[SE], −0.012 [0.13]; *P*=0.89). We conclude that a genetic variant in *PDE3A* influences endothelial function in early life and leads to increased risk of ischemic stroke. Subtle, measurable changes to the vasculature that are influenced by genetics also influence risk of ischemic stroke.

Genome-wide association studies (GWAS) have identified numerous variants underlying cardiovascular diseases including stroke and coronary heart disease.^1,2^ However, in the main, the mechanism of these variants on disease risk has been elusive. A complementary approach to the standard GWAS design of clinical end points such as stroke is to integrate data with GWAS on intermediate phenotypes of disease risk such as carotid intima-media thickness or plaque.^3^ Identifying variation that is common to an intermediate phenotype and disease outcome has the potential to identify mechanism-specific associations with disease and to illuminate causal pathways.

Endothelial function is one such phenotype of importance to stroke.^4^ The endothelium is a group of cells lining blood vessels that functions both as a barrier, controlling the passage of materials into and out from the blood stream, and as a signal transducer, regulating vessel structure and function.^5^ Impaired endothelial function is involved in the initiation of atherosclerosis, as well as latter plaque instability,^6^ and promotion of small vessel arteriopathy.^7,8^ Several techniques exist for measuring endothelial function in vivo.^5^ One validated method, ultrasound flow-mediated dilatation (FMD), measures the response of the arterial endothelium to reactive hyperemia via inflation and subsequent deflation of a blood flow constricting cuff (sphygmomanometer).^5^

Here, we perform a GWAS of FMD in adolescents from ALSPAC (Avon Longitudinal Study of Parents and Children). We integrate the results with a large-scale GWAS of stroke in over 60 341 cases and 452 969 controls (MEGASTROKE)^2^ and use genome-wide colocalization approaches to identify genetic variation that contributes to stroke through impaired endothelial function in adolescents.

## Methods

Data used in this study are available to all researchers through application to ALSPAC (http://www.bristol.ac.uk/alspac/researchers/) and UK Biobank (https://www.ukbiobank.ac.uk/register-apply/). Publicly available GWAS data pertaining to cardiovascular risk factors and traits used in this study are available from http://megastroke.org (stroke), https://www.cardiomics.net/download-data (coronary heart disease), http://www.diagram-consortium.org/downloads.html (type 2 diabetes mellitus), http://www.broadcvdi.org/ (atrial fibrillation), http://lipidgenetics.org/ (lipids), and https://conservancy.umn.edu/handle/11299/201564 (smoking status).

### Study Subjects

Study Populations are outlined in Table 1. Pregnant women resident in Avon, UK with expected dates of delivery April 1, 1991, to December 31, 1992, were invited to take part in ALSPAC—a prospective observational study of the genetic and environmental determinants of development and health from the prenatal period into adulthood.^9–11^ The initial number of pregnancies enrolled was 14 541, which was later increased to 15 247 by retrospective recruitment. A total of 15 656 fetuses were included in further studies, of which 14 889 were alive at 1 year of age. Please note that the ALSPAC study website contains details of all the data that are available through a fully searchable data dictionary and variable search tool (http://www.bristol.ac.uk/alspac/researchers/our-data/). We studied endothelial function in 7557 of these children aged between 9 and 12 years (mean [SD], 10.7 [0.25] years). Age, sex, body mass index, blood pressure, and environmental factors were collected as described previously.^9^ Room and skin temperatures were assessed using a commercial digital thermometer immediately before the vascular examination. Brachial artery endothelial function was successfully measured in 88% of children by FMD.^11^ The right brachial artery was imaged using high-resolution ultrasound (ALOKA 5500) 5 to 10 cm above the antecubital fossa with the probe held in a stereotactic clamp. Edge detection software (Brachial Tools, MIA, IA) was used to measure the brachial artery diameter at 3-second intervals throughout the 11-minute recording protocol. Brachial artery FMD was induced by a 5-minute inflation of a pneumatic cuff to 200 mm Hg, around the forearm immediately below the medial epicondyle followed by rapid deflation using an automatic air regulator (Logan Research, United Kingdom). We used FMD expressed as a percentage calculated using peak diameter in response to reactive hyperemia in relation to the baseline diameter.^12^

**Table 1.**
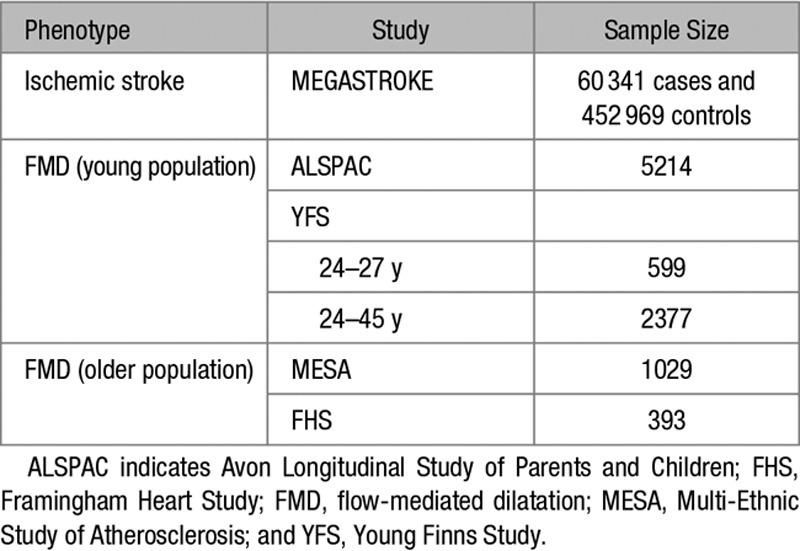
Study Populations

### Genotyping and Quality Control in ALSPAC

We used imputed genotypic data of the ALSPAC. Genotyping, quality control, and imputation has been described previously.^13^ GWAS data were generated by Sample Logistics and Genotyping Facilities at Wellcome Sanger Institute and LabCorp (Laboratory Corporation of America) using support from 23andMe. Imputation was performed using the complete reference panel from the third phase of the 1000 Genomes Project.^14^ Five thousand two hundred ninety-seven children had both genotyping information and FMD measurements. We excluded 83 cryptically related samples with PI-HAT >0.1875, determined using PLINK2,^15^ meaning the final analyses were performed on 5214 samples. We analyzed all single-nucleotide polymorphisms (SNPs) that were polymorphic (minor allele frequency, >1%) in European samples and had an imputation info value >0.5. PLINK2 was used to estimate identity-by-descent and to perform ancestry-informative principal components (PC) analysis.^15^

### MEGASTROKE Genome-Wide Summary Statistics

We used summary statistics, downloaded from http://megastroke.org, derived from MEGASTROKE—a transethnic genome-wide meta-analysis of stroke.^2^ Our primary analysis was for all ischemic stroke cases versus controls (60 341 cases and 452 969 controls). We also explored associations with ischemic stroke subtypes in MEGASTROKE: cardioembolic stroke (9006 cases and 426 629 controls), large artery stroke (6688 cases and 345 446 controls), and small vessel stroke (11 710 cases and 346 101 controls).

### Genome-Wide Analyses in Adolescents From ALSPAC

Per-allele β-coefficients and SEs for FMD were generated in a regression model that included age, sex, room temperature, body temperature, and 8 ancestry-informative PCs. Covariates were selected based on their significant association with FMD in previous ALSPAC publications.^11^ For sensitivity analysis, we performed analysis of a second model that was additionally adjusted for heritable covariates: body mass index, systolic blood pressure, and diastolic blood pressure. All statistical tests conducted were 2 sided, and SNPTEST v2.5.4-beta3 was used to perform the analysis. We used a *P* threshold of 5.0×10^−8^ to determine statistical significance. We discarded significant low-frequency SNPs (minor allele frequency, <0.05) without substantial linkage disequilibrium support (1 region).

### Validation Datasets

We explored validation of the FMD-associated novel SNP in independent cohorts of FMD measured in both younger and older cohorts of subjects.

We sought replication in younger subjects in YFS (Young Finns Study)—a study of young subjects (age, 24–45 years).^16^ The YFS cohort is a Finnish longitudinal population study sample on the evolution of cardiovascular risk factors from childhood to adulthood. In the present study, we used the variables measured in 2001 and described in detail previously.^17^ For these subjects, genotyping was performed in 2009 using a custom-built Illumina Human 670k BeadChip at the Welcome Trust Sanger Institute and imputed to 1000 Genomes phase 3.^18^ We attempted replication of a single SNP in the youngest quartile (age, 24–27 years) and overall (age, 24–45 years). All analyses included age, sex, center, and ancestry-informative PCs as covariates.

We sought replication of the novel SNP with FMD in older individuals of European ancestry in both the FHS (Framingham Heart Study) and the MESA (Multi-Ethnic Study of Atherosclerosis).

The MESA subjects analyzed were 1029: 50% men; mean age, 61 years (SD, 10 years). Flow-mediated dilation (FMD) of the brachial artery mean (SD), 4.8 (3.1). FHS subjects analyzed were 393: 38% men; mean age, 41 years (SD, 8 years). FMD of the brachial artery mean (SD), 5.9 (3.7).

For the MESA cohort, analysis was restricted to subjects with European ancestry based on the self-reported race/ethnicity. MESA and FHS genotype panels are described elsewhere.^19,20^ Standard quality control procedures for the genotype data were performed using PLINK. We excluded SNPs with a minor allele frequency <5% or significant deviation from Hardy-Weinberg equilibrium (*P*<1.0×10^−6^). SHAPEIT and IMPUTE2 software were used to impute PDE3A (Phosphodiesterase 3A) region (chr 12; p12.2:18060181-23060180; GRCh v37), using the 1000 Genomes reference panel (GRCh v37, phase III).^18^ Imputed SNPs with an imputation quality estimate (R^2^<0.40) were removed for association tests. The first 3 PCs for population structures were derived using the smartpca script in EIGENSTRAT and included as covariates in association analyses for both MESA and FHS.^21^

For both cohorts, linear regression analyses assuming an additive model were computed using 2 models: model 1 that included sex, age, and PCs 1 to 3 and model 2 where body mass index, diastolic blood pressure, and systolic blood pressure at the time of the measurements were added to the covariates of model 1. Linear mixed-effects Kinship models (package lmekin in R) for family data were implemented in FHS.

### Further Analysis of Novel Association

We used a Bayesian test for colocalization at the novel gene between FMD GWAS summary results and stroke to assess whether 2 association signals are consistent with a shared causal variant.^22^ The gwas-pw package was used to perform all analyses (https://github.com/joepickrell/gwas-pw).

### Association With Cardiovascular Risk Factors, Outcomes, and Coronary Artery Expression

Using data from publicly available repositories and UK Biobank, we assessed the impact of the *PDE3A* rs11045239 variant on cardiovascular risk factors: systolic blood pressure (id 4080), diastolic blood pressure (id 4079), pulse pressure (derived from the former 2 variables), pulse wave arterial stiffness (id 21021), hypertension (self-reported, id 200002=1465), hypercholesterolemia (self reported: id 20002=1473), ever smoking,^23^ as well as cardiovascular diseases: coronary artery disease,^24^ type 2 diabetes mellitus,^25^ and atrial fibrillation.^26^

UK Biobank (http://www.ukbiobank.ac.uk) is a prospective study that recruited 500 000 community-dwelling participants aged 40 to 69 years from across the United Kingdom between 2006 and 2010. The study collects extensive data from questionnaires, interviews, health records, physical measures, biological samples, and imaging. For all variables we considered in this analysis, we excluded outlier readings that were >3 SDs from the median value.

The UK Biobank genotyping procedure has been described elsewhere.^27^ In short, 2 custom genotyping arrays were used to genotype 49 950 (UK BiLEVE Axiom Array) and 438 427 participants (UK Biobank Axiom Array).^27,28^ Genotype data (805 426 markers) were available for 488 377 individuals and were subsequently imputed to the Haplotype Reference Consortium reference panel (39 131 578 autosomal SNPs). Imputed genotypes were available for 487 442 individuals in this study.^27^ From the resulting imputed dataset, we excluded (1) individuals who did not segregate with European individuals based on PC analysis, (2) individuals with high levels of heterozygosity or missingness (>5%), and (3) individuals whose reported sex did not match with sex inferred from the genetic data.

All UK Biobank analyses included age, sex, and ancestry-informative PC as covariates. In analyses of blood pressure variables, we additionally corrected for body mass index, as has been the convention in GWAS studies.^29^

In addition, we investigated the association of rs11045239, and other variants in close linkage disequilibrium (r^2^>0.8), calculated using LDlink,^30^ with expression of *PDE3A* in coronary arteries from Genotype-Tissue Expression.^31^ All analyses were performed using the Genotype-Tissue Expression portal (https://gtexportal.org/home/; accessed May 14, 2019).

### Standard Protocol Approvals, Registrations, and Patient Consents

Ethical approval for the study was obtained from the ALSPAC Ethics and Law Committee and the Local Research Ethics Committees. UK Biobank received ethical approval from the research ethics committee (reference 11/NW/0382). All participants provided informed consent to participate. The present analyses were conducted under UK Biobank application number 36509.

## Results

We performed a genome-wide association analysis of FMD in 5214 adolescents from ALSPAC. The inflation of test statistic using genomic control was 1.014, while the linkage disequilibrium score regression intercept was 1.001 indicating no inflation of test statistics.^1^ We identified an SNP (rs11045239) on chromosome 12 (12:20579694) that was associated with FMD (β[SE], 0.38 [0.070]; *P*=3.8×10^−8^). The risk allele (A) had a frequency of 40% among Europeans, and the SNP was located in an intron of the *PDE3A* gene, which encodes phosphodiesterase 3A—a member of the cyclic GMP (cGMP)–inhibited cyclic nucleotide phosphodiesterase family.

**Figure 1.**
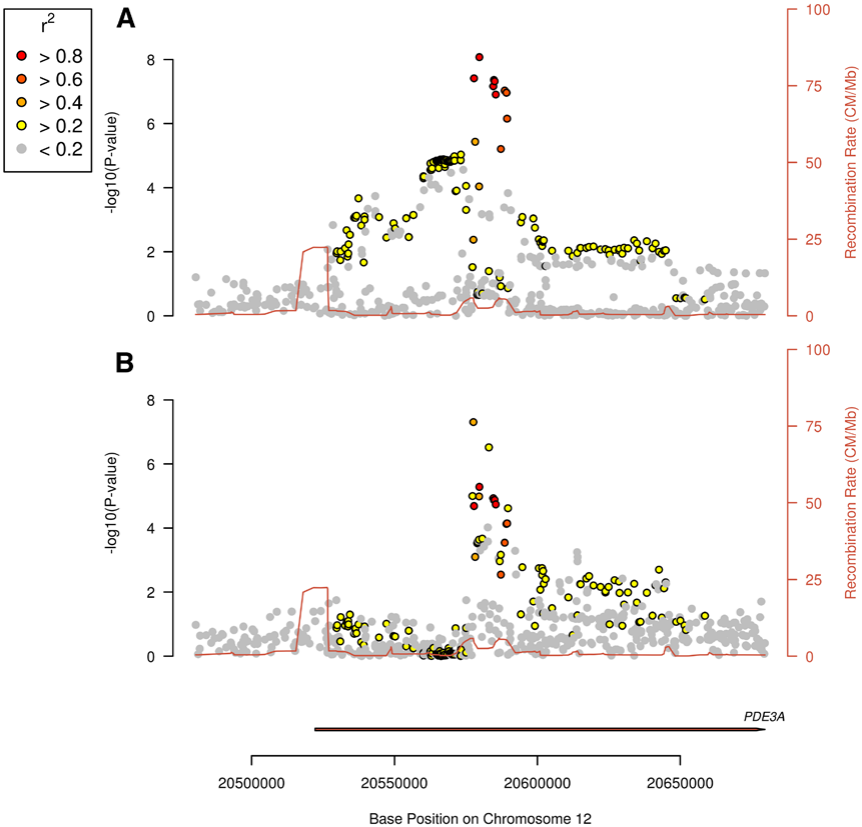
Colocalization of Genetic Associations with Flow Mediated Dilation and Ischaemic Stroke at *PDE3A* Locus. Plots of −log10(*P*) for association of single-nucleotide polymorphisms (SNPs) in the *PDE3A* gene region. Each point indicates an SNP association with the trait, with color indicating correlation (r^2^) with the lead SNP, rs11045239. Red line indicates recombination rate (CM/Mb) at this region of the genome. **A**, SNP associations with flow-mediated dilatation by chromosome position. **B**, SNP associations with ischemic stroke by chromosome position.

To identify regions with evidence of shared genetic variation impacting on FMD and ischemic stroke, we performed a genome-wide Bayesian colocalization analysis.^22^ This analysis highlighted 1 region around the same *PDE3A* gene where the underlying genetic variation was highly likely to be associated with both FMD and ischemic stroke (posterior probability, 97%; Figure 1). The most likely causal SNP based on Bayesian colocalization was rs11045239, although this was not conclusive (posterior probability, 65%). The 95% credible set contained 4 other SNPs: rs12811752, rs11045244, rs7489190, and rs10841519. The same *PDE3A* locus was recently reported to be associated at genome-wide significance with ischemic stroke in MEGASTROKE (lead SNP, rs7304841; odds ratio, 1.05 [95% CI, 1.03–1.07]; *P*=4.9×10^−8^).^2^ Effects were similar across ischemic stroke subtypes: cardioembolic stroke (odds ratio, 1.04 [95% CI, 1.00–1.08]), large artery stroke (odds ratio, 1.05 [95% CI, 1.00–1.10]), and small vessel stroke (odds ratio, 1.05 [95% CI, 1.01–1.08]). No other regions were highlighted with posterior probability of shared genetic effects >50%.

Having established that genetic variation in a single locus in *PDE3A* is associated with both ischemic stroke and FMD, we sought validation of the association in other cohorts with FMD data. As no other cohorts with genetic data and FMD measurement in adolescents exist to our knowledge, we sought replication in adult populations. In the YFS, we first analyzed the youngest individuals (the first quartile, 24–27 years; n=599) who most closely resemble the discovery cohort. In these individuals, there was evidence at nominal significance that the variant was associated with disease (β[SE], 0.47 [0.23]; *P*=0.047). Conversely, when considering the whole cohort (age, 24–45 years; n=2337), we did not observe a significant effect (β[SE], 0.14 [0.13]; *P*=0.25). We also explored whether the same variant was associated with FMD in older individuals from the FHS and MESA (n=1186). We found no evidence of an association in this older group (β[SE], −0.012 [0.13]; *P*=0.89). These results might indicate that the genetic variant identified in the study has different influence on endothelial function in adolescents compared with adults.

**Figure 2.**
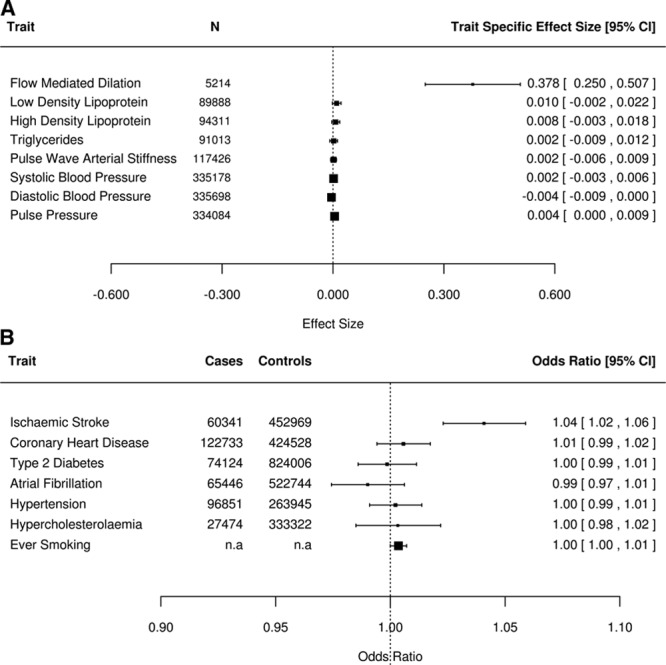
Associations with *PDE3A* rs11045239 single-nucleotide polymorphism. Plot of the association of each risk allele (effect size and 95% CI) of the *PDE3A* rs11405239 single- nucleotide polymorphism on (**A**) cardiovascular risk factors and (**B**) cardiovascular disease outcomes and binary risk factor traits. Exact numbers of cases and controls were not available in publicly available data for ever smoking.

To establish whether variation in *PDE3A* was associated solely with endothelial function, or influenced other cardiovascular pathways, we looked at association of the *PDE3A* rs11045239 SNP with other cardiovascular risk factors and outcomes in large publicly available datasets and UK Biobank. The *PDE3A* rs11045239 variant was not associated with any other related cardiovascular traits or outcomes (all *P*>0.05; Figure 2), suggesting the association with ischemic stroke is not mediated through alternative cardiovascular pathways.

In addition, we looked at the association of *PDE3A* SNP rs11045239 and its proxies with mRNA expression of *PDE3A* in coronary arteries from Genotype-Tissue Expression. Although rs11045239 was not significantly associated with *PDE3A* expression (*P*=0.075), all proxy SNPs showed association at *P*<0.05 (Table 2). The A allele association with increased FMD and risk of stroke was associated with lower levels of *PDE3A.* Therefore, although not conclusive, this finding is consistent with variants in the *PDE3A* locus influencing expression of *PDE3A* in relevant tissues.

**Table 2.**
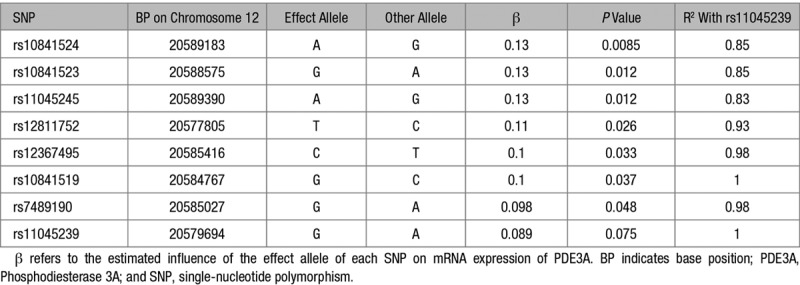
Association of SNPs in Linkage Disequilibrium With rs11045239 With mRNA Expression of *PDE3A* in Coronary Arteries From Genotype-Tissue Expression (n=152)

## Discussion

We identified a genetic variant in the *phosphodiesterase 3A* gene that was associated at genome-wide significance with both FMD in early life and ischemic stroke. Colocalization analyses indicated that the same genetic variation in *PDE3A* associated with ischemic stroke is also associated with FMD, thereby showing the shared association is not merely coincidental. The association with variants in *PDE3A* was consistent across all stroke subtypes, which are presumed to have distinct etiologies. It, therefore, seems probable that the variant acts via a risk factor common to all subtypes. Exploring association of the same genetic variant with multiple other cardiovascular risk factors and outcomes, we could find no association that the variant also acts via other independent processes. There was some suggestion that variants at the *PDE3A* locus influence expression of *PDE3A* in coronary arteries, although this was not conclusive. An interpretation consistent with these data is that the associated genetic locus influences expression of *PDE3A* in arterial tissues, which leads to altered endothelial function and subsequent risk of ischemic stroke. Mediation analysis might help to shed light on whether this is a potential causal pathway.

Although it is not possible to determine the exact mechanism by which the *PDE3A* variant confers risk of ischemic stroke and alters endothelial function without further experimental data, some speculation on potential pathways is warranted. In vascular smooth muscle, phosphodiesterases play a key role in the NO/cGMP pathway—one of the most important regulators of vascular smooth muscle contraction and platelet activation. NO activates sGC (soluble guanylyl cyclase), which activates cGMP, activating many signaling molecules, in particular, PRKG (protein kinase G), which in turn promotes vascular smooth muscle contraction. Phosphodiesterases decompose cGMP, as well as cAMP, into GMP and AMP, respectively. This has the effect of monitoring the influence of cGMP on downstream processes such as smooth muscle contraction and platelet activation. Inhibition of phosphodiesterases has, therefore, been a target of multiple pharmaceuticals, with the intended effect of prolonging the influence of cGMP and promoting vasodilation. One such pharmaceutical cilostazol is a selective inhibitor of phosphodiesterase type 3 and is used primarily to treat peripheral vascular disease. Studies in East Asian populations suggest it ameliorates endothelial dysfunction and reduces recurrent stroke rates in patients with previous stroke,^32^ although not all studies have been positive,^33^ and it is currently being trialed for use to prevent recurrence after lacunar stroke in European populations.^34^ Off-label studies have also suggested it has the potential to prevent progression of intracranial arterial stenosis.^35^ However, *PDE3A* is also expressed in cardiac muscle, and whether the variant might influence stroke risk (particularly of cardioembolic source) via altered expression in the heart cannot be ruled out.

Why we detected an association of the *PDE3A* locus with endothelial function in adolescents, but not in adults, deserves further consideration. Aging has a considerable impact on endothelial biology^36,37^; normal endothelial function in children has a different molecular basis to the (dysfunctional) endothelium of older adults. In particular, decreases in NO production, expression of endothelial NO synthase,^38^ expression of cell adhesions molecules such as ICAM1 (intercellular adhesion molecule 1) and growth factors such as VEGF (vascular endothelial growth factor), as well as increases in levels of endothelin with age, contribute significant changes to endothelial biology. Whether these factors lead to a fundamental change in the relationship between the *PDE3A* locus and endothelial function, or simply influence the signal-to-noise ratio and, therefore, our ability to detect an effect, remains unclear. Nevertheless, the fact that the same *PDE3A* locus has a subsequent influence on stroke risk suggests that the changes that occur in adolescence have a lasting impact on the vasculature and subsequent pathological processes.

Our study has limitations. We demonstrate that genetic variation that influences levels of *PDE3A* influences endothelial function and subsequently, ischemic stroke. As such, we demonstrate a pathway by which risk of stroke conferred. However, we cannot rule out other horizontal pathways by which the *PDE3A* locus also leads to risk of ischemic stroke. However, we note that our analysis of other cardiovascular risk factors showed no association. As we were not able to assess endothelium-independent vasodilation in response to a nitrate, we cannot be absolutely conclusive that our FMD measure results reflect endothelium-dependent vasodilation. Rather, it is possible that they reflect nonspecific alterations in vascular reactivity.

## Perspectives

In conclusion, we show that genetic variation in phosphodiesterase 3A leads to altered endothelial function in early life, mostly likely via expression of *PDE3A*, and also leads to increased risk of ischemic stroke, thus elucidating a pathway by which risk of disease is likely conferred.

## Acknowledgments

We are extremely grateful to all the families who took part in ALSPAC (Avon Longitudinal Study of Parents and Children), the midwives for their help in recruiting them, and the whole ALSPAC team, which includes interviewers, computer and laboratory technicians, clerical workers, research scientists, volunteers, managers, receptionists, and nurses. Data used in this work were obtained from UK Biobank (data application 36509). We are grateful to UK Biobank for making the data available and to all UK Biobank study participants, who generously donated their time to make this resource possible. H.S. Markus and M. Traylor conceived the study and obtained funding. M. Traylor and A. Amin Al Olama designed the analysis plan; M. Traylor, A. Amin Al Olama, S. Marini, J. Chung, and L.-P. Lyytikäinen performed the statistical analyses. M. Traylor and A. Amin Al Olama wrote the first draft of the manuscript. L.-P. Lyytikäinen, T. Lehtimäki, and O.T. Raitakari contributed data. All authors read and approved the final manuscript.

## Sources of Funding

This study was supported by the European Union’s Horizon 2020 research and innovation programme under grant agreement No. 667375. The UK Medical Research Council and Wellcome (grant reference No. 102215/2/13/2) and the University of Bristol provide core support for ALSPAC (Avon Longitudinal Study of Parents and Children). This publication is the work of the authors, and M. Traylor and H.S. Markus will serve as guarantors for the contents of this article. This work was supported by a British Heart Foundation Programme Grant (RG/16/4/32218). H.S. Markus is supported by a National Institute for Health Research (NIHR) Senior Investigator award, and his work is supported by the Cambridge Universities NIHR Comprehensive Biomedical Research Centre. C.D. Anderson is supported by National Institutes of Health R01NS103924 and K23NS086873. YFS (Young Finns Study) has been financially supported by the Academy of Finland: grants 286284, 134309 (Eye), 126925, 121584, 124282, 129378 (Salve), 117787 (Gendi), and 41071 (Skidi); the Social Insurance Institution of Finland; Competitive State Research Financing of the Expert Responsibility area of Kuopio, Tampere and Turku University Hospitals (grant X51001); Juho Vainio Foundation; Paavo Nurmi Foundation; Finnish Foundation for Cardiovascular Research; Finnish Cultural Foundation; Sigrid Juselius Foundation; Tampere Tuberculosis Foundation; Emil Aaltonen Foundation; Yrjö Jahnsson Foundation; Signe and Ane Gyllenberg Foundation; Diabetes Research Foundation of Finnish Diabetes Association; EU Horizon 2020 (grant 755320 for TAXINOMISIS); European Research Council (grant 742927 for MULTIEPIGEN project); and Tampere University Hospital Supporting Foundation. Availability of data and materials: ALSPAC: http://www.bristol.ac.uk/alspac/researchers/; MEGASTROKE GWAS: http://megastroke.org; Coronary Artery Disease GWAS: https://www.cardiomics.net/download-data; Type 2 Diabetes GWAS: http://www.diagram-consortium.org/downloads.html; Atrial Fibrillation GWAS: http://www.broadcvdi.org/; UK Biobank: https://www.ukbiobank.ac.uk/; Smoking Status: https://conservancy.umn.edu/handle/11299/201564.

## Disclosures

C.D. Anderson has consulted for ApoPharma, Inc. The other authors report no conflicts.

## Novelty and Significance

### What Is New?

- Using a large-scale genetic study of stroke (MEGASTROKE) and a study of endothelial function in adolescents (ALSPAC [Avon Longitudinal Study of Parents and Children]), we identified a genetic locus in the *PDE3A* gene, encoding phosphodiesterase 3A, that was associated with both endothelial function and ischemic stroke. The genetic variant was not associated with any other cardiovascular risk factor suggesting its influence on stroke acts solely through altered endothelial function.

### What Is Relevant?

- Our results highlight a pathway that in adolescence is influenced by genetic variation, which influences risk of stroke in later life.

### Summary

Genetic variation in *PDE3A* influences risk of stroke through altered endothelial function, as measured by flow-mediated dilatation.
